# Novel *CHM* mutations in Polish patients with choroideremia – an orphan disease with close perspective of treatment

**DOI:** 10.1186/s13023-018-0965-5

**Published:** 2018-12-12

**Authors:** Anna Skorczyk-Werner, Anna Wawrocka, Natalia Kochalska, Maciej Robert Krawczynski

**Affiliations:** 1 Department of Medical Genetics, Poznan University of Medical Sciences, 8, Rokietnicka St, 60-806 Poznan, Poland; 2 Centers for Medical Genetics GENESIS, 4, Grudzieniec St, 60-601 Poznan, Poland

**Keywords:** Choroideremia (CHM), *CHM* gene, Rab escort protein 1 (REP-1), New mutations

## Abstract

**Background:**

Choroideremia (CHM) is a rare X-linked recessive retinal dystrophy characterized by progressive chorioretinal degeneration in the males affected. The symptoms include night blindness in childhood, progressive peripheral vision loss and total blindness in the late stages. The disease is caused by mutations in the *CHM* gene encoding Rab Escort Protein 1 (REP-1). The aim of the study was to identify the molecular basis of choroideremia in five families of Polish origin.

**Methods:**

Six male patients from five unrelated families of Polish ethnicity, who were clinically diagnosed with choroideremia, were examined in this study. An ophthalmologic examination performed in all the probands included: best-corrected visual acuity, slit-lamp examination, funduscopy, fluorescein angiography and perimetry. The entire coding region encompassing 15 exons and the flanking intronic sequences of the *CHM* gene were amplified with PCR and directly sequenced in all the patients.

**Results:**

Five variants in the *CHM* gene were identified in the five families examined. Two of the variants were new: c.1175dupT and c.83C > G, while three had been previously reported.

**Conclusions:**

This study provides the first molecular genetic characteristics of patients with choroideremia from the previously unexplored Polish population.

## Background

Choroideremia (CHM, MIM 303100) is a rare X-linked recessive retinal dystrophy leading to degeneration of the retinal pigment epithelium, photoreceptors and choroid. Affected males develop night blindness in late childhood, progressive loss of peripheral visual fields, and loss of central visual acuity in the late stage of the disease. Typically, female carriers are asymptomatic, but funduscopy often reveals patchy areas of chorioretinal atrophy, although fully affected females have been also described [1–4]. The prevalence of choroideremia is estimated to be 1 in 50,000 [1]. Choroideremia is caused by mutations in the *CHM* gene, encoding Rab Escort Protein 1 (REP-1). The *CHM* gene is located on chromosome X at position Xq21.2. The gene spans over 150 kb and consists of 15 exons [5].

Rab Escort Protein 1 (REP-1) is an essential component of the Rab geranylgeranyltransferase enzyme (RGGTase) II complex that mediates correct intracellular vesicular transport [1]. REP-1 encodes a molecular chaperone for small guanosine triphoshatases (GTPases) from the Rab family, transporting them to Rab geranylgeranyltransferase. The enzyme enables prenylation, lipid modification of Rab proteins crucial for intracellular vesicular trafficking process. Prenylated Rabs are then delivered to their target membranes by REP1, thus in the absence of REP1 unprenylated Rabs are accumulated in cytosol [6, 7].

Altogether 280 disease-associated variants in the *CHM* gene including substitutions, small insertions and deletions, large deletions ranging from single exons to whole gene and splice defects have been reported to date in patients with choroideremia. Most of the pathogenic variants identified in the *CHM* gene are loss-of-function mutations that abolish functional REP-1 [8–12]. The lack of REP-1 is compensated by REP-2 in all tissues, excluding the retina. REP-1 is essential for the function of the RPE (retinal pigment epithelium) and photoreceptors. The absence of REP-1 is the cause of disruption to normal intracellular trafficking in the retina and thereby retinal degeneration in choroideremia [13].

## Materials and methods

### Clinical diagnosis

Six male patients from five unrelated families of Polish ethnicity who were clinically diagnosed with choroideremia were examined in this study. Opthalmologic examinations including best-corrected visual acuity (BCVA), slit-lamp biomicroscopy, funduscopy, fluorescein angiography (FA) and perimetry (automated visual field testing) were done in all the probands. Electroretinography (ERG) was performed in two patients: Patient 3 and Patient 6, and Optical Coherent Tomography (OCT) in Patient 2 and Patient 3.

### Molecular genetic analysis

Blood samples from the individuals affected were obtained for genetic examination. Genomic DNA was extracted from peripheral blood using the conventional salting-out procedure. The entire coding region encompassing 15 exons and flanking intronic sequences of the *CHM* gene was amplified with PCR and directly sequenced in all the patients. The primers used for amplification and sequencing, as well as the PCR conditions are available upon request. The polymerase chain reaction (PCR) products were purified with the use of ExoSAP-IT (Exonuclease I and Shrimp Alkaline Phosphatase Cleanup for PCR products, Affymetrix) and directly sequenced using Dye Terminator chemistry (v3.1BigDye® Terminator, Life Technologies). The sequencing products were separated on an ABI 3130xl capillary sequencer (Applied Biosystems). The sequences obtained were verified by comparing them to the reference sequence of the *CHM* gene (GenBank NM_000390.2) and screened for mutations. Any variations identified were referred to the Human Gene Mutation Database (HGMD), Exome Variant Server (EVS), ExAC Browser Beta (Exome Aggregation Consortium 2015) and gnomAD browser beta (genome Aggregation database) for the *CHM* gene*.* Novel variants identified in this study were classified according to the American College of Medical Genetics and Genomics (ACMG) guidelines.

## Results

**Fig. 1:**
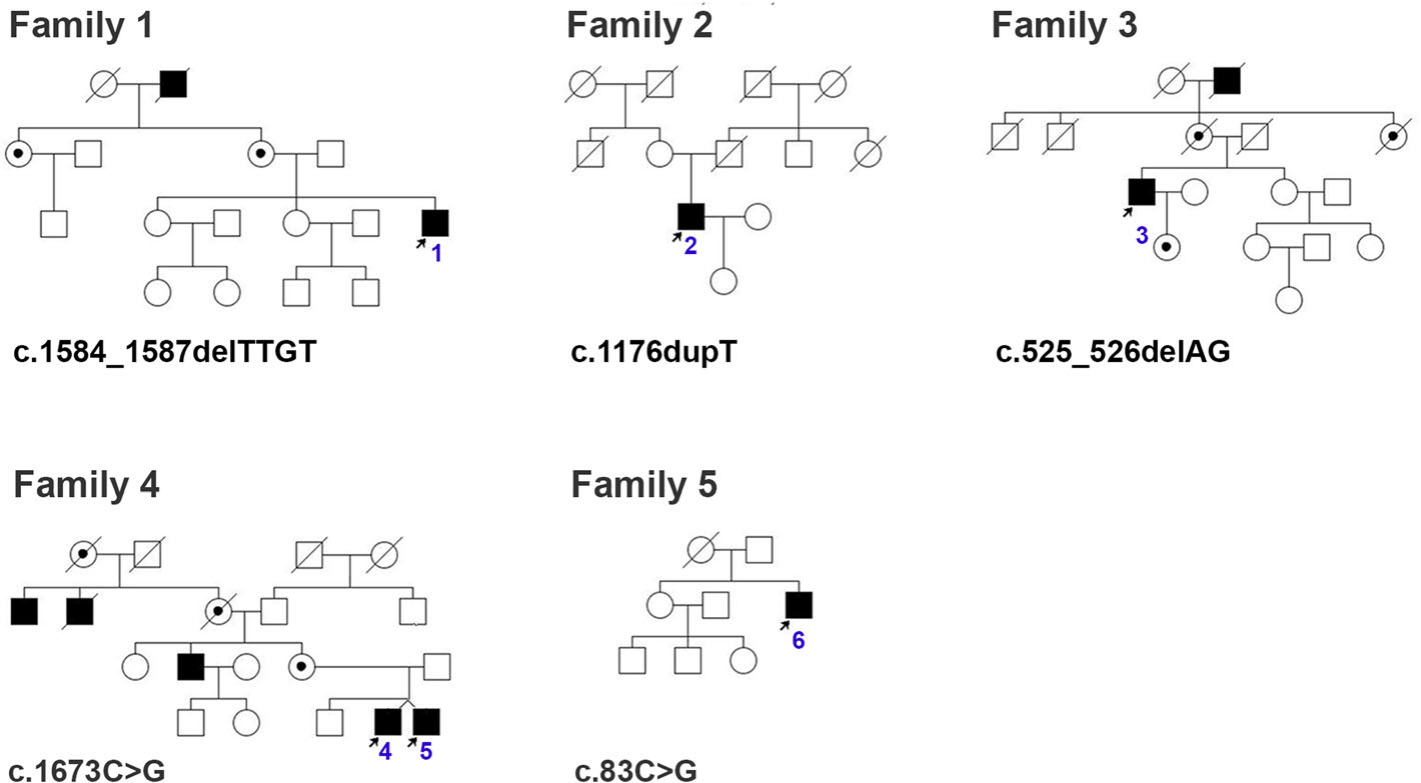
Pedigrees of the families with *CHM* mutations. Filled symbols indicate individuals affected with choroideremia and unfilled symbols indicate unaffected individuals. Dotted circles indicate female carriers. A slash indicates a deceased person. Arrows indicate probands. Blue digits indicate patient numbers

Six male patients aged 29–67 exhibiting typical signs of choroideremia, including night blindness and progressive loss of peripheral visual fields, were examined. Reduced visual acuity was observed in all but one patient. Five different variants in the *CHM* gene, including two novel and three previously described mutations, were identified in five unrelated families. Pedigrees of these families are shown in Fig. 1.

**Fig. 2:**
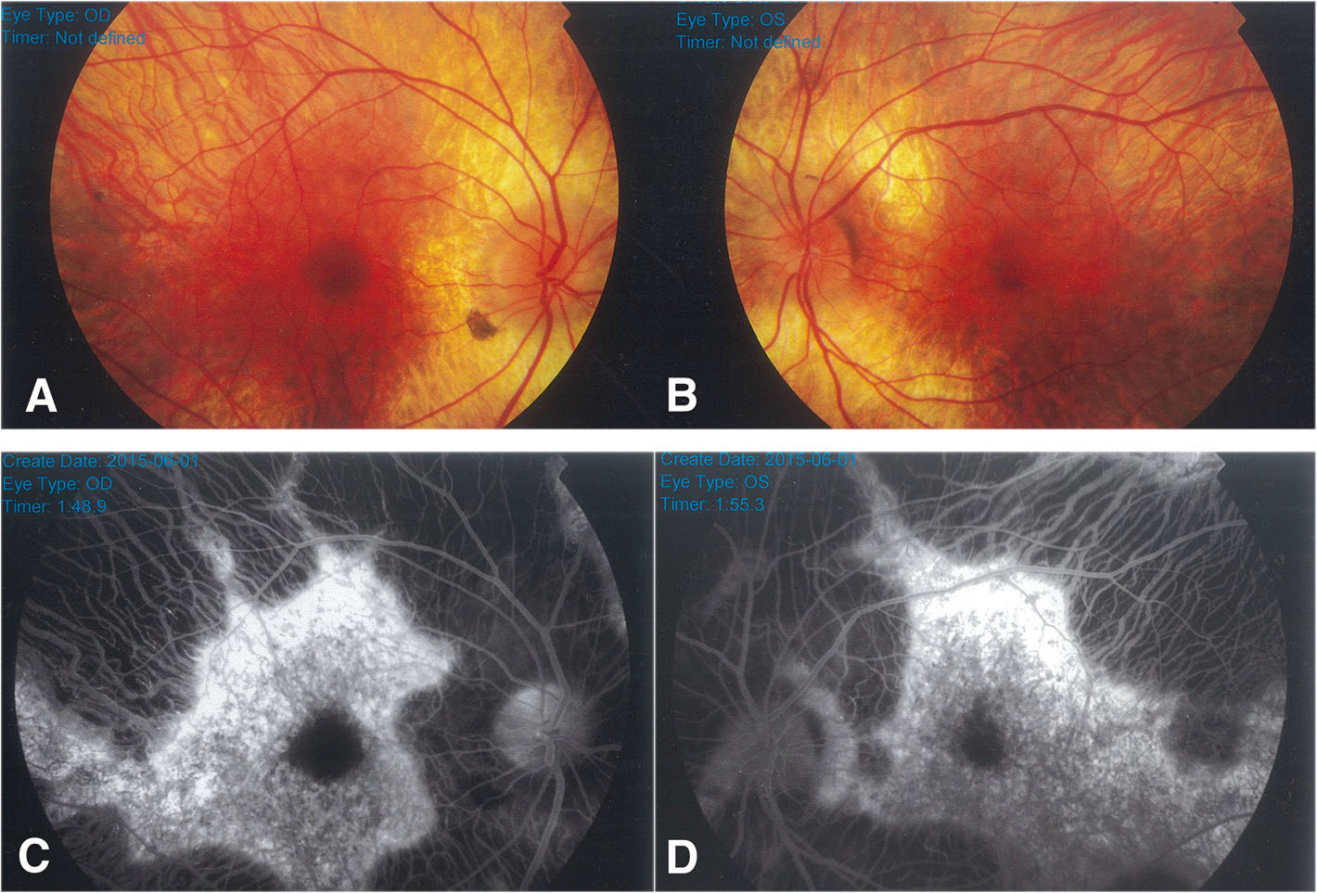
Retinal features of Patient 1. **a** fundus photograph of the right eye; **b** fundus photograph of the left eye; **c** fluorescein angiography of the right eye; **d** fluorescein angiography of the left eye

Patient 1 was a 29-year-old man who was referred to the genetics clinic at the age of 27 due to mildly reduced visual acuity (0.8–0.9) and disorders of night vision. Ophthalmological examination revealed irregular loss of visual field and diffuse loss of RPE and choriocapillaris (Fig. 2). Visual field was reduced to 20°. Similar clinical symptoms were observed in the patient’s maternal grandfather (Fig. 1). DNA sequencing of the coding region of the *CHM* gene revealed a previously described mutation c.1584_1587delTTGT in exon 13 (p.Val529Hisfs*7).

**Fig. 3:**
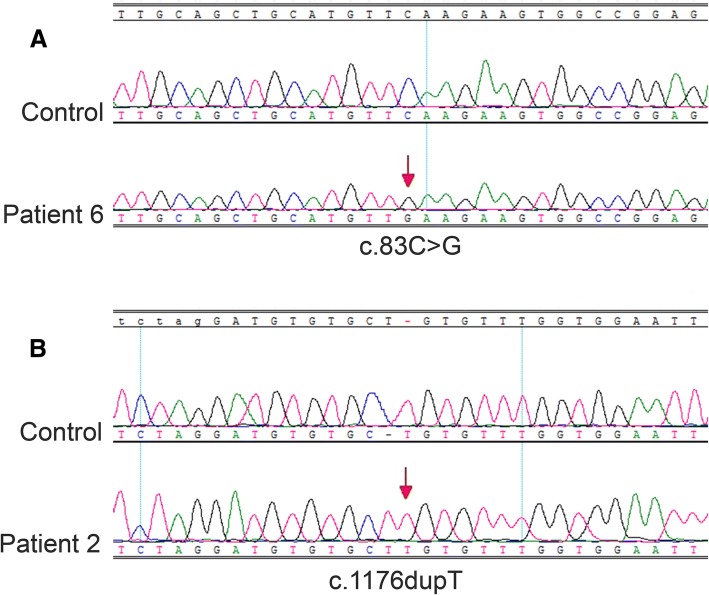
Chromatograms of the *CHM* novel variants: **a** the upper chromatogram shows a wild-type sequence in a control individual, the lower chromatogram shows c.83C > G identified in Patient 6, **b** the upper chromatogram: control, the lower electropherogram: c.1176dupG in Patient 2. Arrows indicate nucleotides that have been changed

Patient 2 was a 37-year-old man who experienced night blindness when he was 10 years old and was diagnosed with retinitis pigmentosa (RP) when he was 18 years old. His best-corrected visual acuity was 0.3 in his left eye, while the visual acuity of his right eye was only slightly reduced (0.8). A visual field examination revealed a loss of peripheral and paracentral vision. Funduscopy and fluorescein angiography (FA) showed a widespread loss of RPE and choriocapillaris. The latest examinations, FA and OCT revealed a complete loss of RPE. The patient is the only child of healthy, unrelated parents, who have no ophthalmologic problems nor a negative family history (Fig. 1). Molecular analysis of the *CHM* gene revealed a novel variant in exon 9, a duplication of one nucleotide c.1176dupT, causing a frameshift that results in premature stop codon p.(Val393Cysfs*25) (Fig. 3). Unfortunately, the suspected de novo origin of the duplication cannot be confirmed by the segregation analysis of the variant, as the proband’s mother is unavailable for analysis.

Patient 3 – a 68-year-old man was referred to the genetics clinic at the age of 58 due to night blindness. Ophthalmological examination revealed reduced visual acuity, hyperopic astigmatism and tunnel vision. Funduscopy showed a diffuse loss of RPE and choriocapillaris. Fluorescein angiography was impossible to perform due to kidney failure. ERG revealed reduced photopic and scotopic responses, while OCT showed minor cystic changes in the macula. Similar symptoms were observed in the patient’s maternal grandfather (Fig. 1). DNA sequencing of the *CHM* gene revealed a deletion of two nucleotides in exon 5: c.525_526delAG (p.Glu177Lysfs*6).

Two 37-year-old men – dizygotic twin brothers - were referred to the genetics clinic 3 years ago due to visual field constriction (visual field reduced to 10–25°). Only one of the brothers manifested night blindness, but funduscopy and fluorescein angiography showed identical results in both twins: diffuse loss of RPE and choriocapillaris. Similar ocular phenotypes were reported in three of patients’ mother’s relatives: a 55-year-old uncle – the brother of their mother, who is unfortunately not available for examination and two deceased grandmother’s brothers. A known mutation: c.1673C > G (p.Ser558*) in exon 14 of the *CHM* gene has been identified in both brothers.

Patient 6 – a 52-year-old man has suffered from reduced night vision since he was at primary school. When he was referred to the genetics clinic, he complained of night blindness, photophobia and myopic astigmatism. Funduscopy revealed a loss of RPE and choroid out of fovea and bone-spicules pigment deposits in the periphery. The visual field was constricted to less than 5° and ERG revealed residual responses. The patient is an isolated case. The parents, the older sister of the patient, and the other members of his family had no ophthalmologic problems. Initially, the proband was diagnosed with choroideremia versus retinitis pigmentosa, but the molecular analysis of the *CHM* gene sequence revealed a novel substitution in exon 2 c.83C > G (p.Ser28*) Fig. 3. The negative family history suggests the de novo origin of the variant, although unfortunately, it is not possible to confirm this supposition as the patient’s mother died a few months ago.

None of the novel variants was found in a control cohort annotated in the Human Gene Mutation Database (HGMD) nor in the Exome Variant Server (EVS) database, the ExAC Browser Beta (Exome Aggregation Consortium 2015) and gnomAD browser beta (genome Aggregation database), which allowed us to exclude these variants being polymorphisms.

## Discussion

In this study, we present the results of the molecular screening in five Polish families suffering from choroideremia. Notably, this is the first report on the *CHM* gene variants causing choroideremia in the Polish population. We identified the molecular background of the disease in all the families, including two novel variants in two families.

In Patient 1 we identified a known variant in the *CHM* gene: c.1584_1587delTTGT (p.Val529Hisfs*7). This deletion has previously been reported in several patients suffering from choroideremia [14–17]. A deletion in exon 5 of the *CHM* gene: c.525_526delAG (p.Glu177Lysfs*6) identified in Patient 3 has also been reported a few times in the literature [5, 18, 19]. The c.1673C > G (p.Ser558*) substitution found in the *CHM* gene of the twins (Patients 4 and 5) has previously been reported in two patients from one family [20].

The c.1176dupT duplication identified in Patient 2 is a frameshift variant p.(Val393Cysfs*25) that has not been reported before. The c.83C > G, p.(Ser28*) nonsense variant identified in Patient 6 is also novel. Although these variants were not functionally tested for potential pathogenicity, both of them resulted in premature stop codon, probably causing the production of a very short non-functional CHM protein. In accordance with the Genetic Variant Interpretation Tool, both novel variants identified in this study have been classified as pathogenic - ‘pathogenic Ib’ [21].

No phenotype-genotype correlations have been found for the variants identified in this study, nor for any of the variants in the *CHM* gene reported to date [11, 12]. Based on the large choroideremia dataset, Freud et al. suggested that the age of visual field constriction and the visual acuity deterioration are not related with the type of *CHM* mutation. They indicated that the critical age for loss of visual acuity was 40 years [11].

A diagnosis of choroideremia can be made clinically, based on a fundus examination and family history, although genetic analysis is indispensable to confirm the diagnosis of CHM. Based on clinical features there are at least two inherited disorders that can be confused with this chorioretinal dystrophy. Some symptoms of retinitis pigmentosa, like night blindness and loss of peripheral visual field, are similar to those of choroideremia, and fundus appearance at later stages of choroideremia can be also similar to that observed at the end stages of RP. However, the degree of migration of pigment into the retina typical for RP is not observed in individuals with choroideremia. Gyrate atrophy of the choroid and retina also can be misdiagnosed with CHM, due to night blindness and chorioretinal atrophy that become widespread during the second and third decade of life, as in choroideremia. A distinguishing feature of these two disorders is type II muscle fiber atrophy. Myopathy is typical for gyrate atrophy of the choroid and retina, but is not observed in choroideremia, although patients suffering from gyrate atrophy very often have no muscle symptoms. Moreover, patients suffering from gyrate atrophy of the choroid and retina have an elevated plasma concentration of ornithine, which is not seen in individuals with CHM. The disease is transmitted according to the AR mode of inheritance, in contrast to XR inheritance in patients with choroideremia [1].

The identification of a pathogenic variant in the *CHM* gene allows the diagnosis of choroideremia to be confirmed [1]. As choroideremia is a non-heterogenous condition, the molecular analysis is not complicated. More than 60% variants are single nucleotide substitutions, small deletions and insertions or small indels located in the coding part of the *CHM* gene [8, 9] therefore in most cases the analysis can be limited to bi-directional sequencing of coding sequence, encompassing 15 exons and exon-intron boundaries. This molecular test should be performed as a mainstay analytical method. A MLPA (Multiplex Ligation-dependent Probe Amplification) assay designed to test for larger deletions and duplications within the *CHM* gene is useful in cases unsolved with Sanger sequencing.

Immunoblot analysis with anti-REP1 antibody is usually performed to validate the absence of CHM protein in the peripheral blood lymphocytes of patients with loss-of-function mutations in the *CHM* gene. Moreover, immunoblot analysis was also suggested as an alternative diagnostic method (to Sanger sequencing) to simply confirm the clinical diagnosis of choroideremia due to the fact that almost all *CHM* variants involve loss of function mutations, resulting in the absence of REP-1 [22].

Lately, there have been reports of some patients with mutations in the *CHM* gene identified using NGS (Next Generation Sequencing) panel for inherited retinal dystrophies [23] or even WES (Whole Exome Sequencing) [16, 24] as a molecular method of choice. Sanger sequencing of the *CHM* coding sequence, alternatively MLPA assay in combination with non-invasive fundus imaging performed and analyzed by experienced ophthalmologists, is usually sufficient to make a diagnosis of choroideremia. It is worth emphasizing the importance of cooperation between ophthalmologists and geneticists in making a diagnosis. Therefore, in most cases there is no need to involve expensive and labour-consuming molecular tests, which are still NGS-based methods. However, in patients with atypical fundus appearance, when there is a problem with clinical distinguishing between choroideremia and retinitis pigmentosa or other retinal dystrophies, NGS-based methods should be involved to make a diagnosis. Advanced methods such as WGS (Whole Genome Sequencing) may be required in some unsolved cases of choroideremia, as deep intronic mutations in the *CHM* gene, which failed to be detected with conventional techniques, cannot be excluded [25]. Moreover, genomic rearrangements such as X-autosome translocations can also occur in patients with choroideremia, although these are detected in patients with additional non-ocular symptoms [19, 26].

Proper and precise molecular diagnostics is particularly important, considering the fact that the possibility of treatment with gene therapy method has emerged recently. Choroideremia is the second human monogenic retinal disorder tested for ocular gene therapy. There are two categories of genetic therapies for retinopathies: mutation-specific gene therapies and therapies that are independent of the type of genetic defect [12, 27].

Therapy with translational read-through-inducing drugs (TRIDs) is an example of mutation-dependent therapy tested in cases with nonsense mutations. TRIDs promote ribosomal misreading of premature stop codons, which results in the incorporation of a near-cognate amino acid to produce a full-length protein [28]. Ataluren (PTC124) is one of the read-through promoting drugs. PTC124 was previously tested on a zebrafish model of CHM, due to a nonsense (UAA) mutation. In zebrafish mutant embryos, ataluren increased survival, prevented the onset of retinal degeneration and corrected the prenylation defect [29]. PTC124 was also tested on human fibroblasts from patients with choroideremia and on RPE cells derived from patient induced pluripotent stem cells (iPSC). In human cells treated with ataluren, the recovery of prenylation activity was observed, although an increase in REP1 protein was not detected [28, 29]. Torriano et al. suggest that the PTC124 efficiency may depend on the conservation and type of target amino acid and its localization, therefore a personalized approach is needed and in vitro screening of the patients’ cells should be considered before including a patient in a clinical trial [28].

Mutation-independent therapies have not been trialled in CHM, but several clinical trials of retinal gene replacement therapy are in progress [30]. Recently, the 24-month results of two clinical trials in male patients treated with sub-foveal adeno-associated viral vector expressing REP1 (AAV2.REP1) have been reported [31, 32]. The two-year results of phase 1 AAV2-mediated therapy showed a serious advert event in one patient who experienced loss in central macular function after treatment and a decline in the area of remaining functional RPE in the treated and untreated eyes at the same rate in all subjects [31].

The 24-month results of phase 2 revealed a sustained improvement in visual acuity in some patients or maintenance of visual acuity. Moreover, this study indicated enhanced safety of the automated sub-foveal injection of the high dose AAV2 REP1, which was guided by real-time Intraoperative OCT [32].

## Conclusion

To conclude, this is the first report of molecular analysis of the *CHM* gene in Polish patients suffering from choroideremia. Our study expands the mutational spectrum of *CHM* mutations, as we also report two novel variants in the *CHM* gene. Considering the fact that genetic therapy approaches can be individualized, in reports on novel mutations in the *CHM* gene even single variants are still not without significance.

## Abbreviations

AAV vector: Adeno-associated viral vector
ACMD: American College of Medical Genetics and Genomics
BCVA: Best-corrected visual acuity
CHM: Choroideremia
ERG: Electroretinography
EVS: Exome Variant Server
ExAC: Exome Aggregation Consortium 2015
FA: Fluorescein angiography
gnomAD: Genome Aggregation database
GTPases: Guanosine triphoshatases
HGMD: Human Gene Mutation Database
iPSC: Induced pluripotent stem cells
LOVD: Leiden Open Variation Database
MLPA: Multiplex Ligation-dependent Probe Amplification
NGS: Next Generation Sequencing
OCT: Optical Coherent Tomography
PCR: Polymerase chain reaction
REP-1: Rab Escort Protein 1
RGGTase: Rab geranylgeranyltransferase
RP: Retinitis pigmentosa
RPE: Retinal pigment epithelium
WES: Whole Exome Sequencing
WGS: Whole Genome Sequencing
